# Toxoplasma encephalitis among AIDS patients in a tertiary care hospital in Mangalore, India

**DOI:** 10.1186/1742-4690-9-S1-P150

**Published:** 2012-05-25

**Authors:** Anand Venugopal, Basavaprabhu Achappa Unnikrishnan, B Deepak Madi, Vishak Surendra, John T Ramapuram

**Affiliations:** 1 Kasturba Medical College, Mangalore, India

## Background

Toxoplasmosis associated with HIV infection is caused by reactivation of a chronic infection and manifests primarily as toxoplasmic encephalitis. This study was done to determine clinical presentations and outcomes of CNS toxoplasmosis and to find out their association with CD4 counts at time of diagnosis and initiation of ART.

## Materials and methods

Retrospective study done by reviewing medical records of HIV-positiveS diagnosed with toxoplasmosis from Jan 2000 to Dec 2010. Diagnosis was based on clinical features, demonstration of elevated IgG by ELISA and associated CT findings. Data obtained was correlated with CD4 count and whether or not patient was on ART. Analysis was done using SPSS version 11.5.

## Results

2826 HIV positives attended Infections Disease Cell from 2000 –2010, of which 33 (1.12%) had CNS Toxoplasmosis. Among 33 cases, 29 were males (88%) and 4 females (12%). Mean age was 37.33 yrs. 10 cases (30.3%) had CNS toxoplasmosis as the initial manifestation of HIV. Most common clinical presentations were fever(58%) and headache (52%). Mean CD4 at diagnosis of toxoplasmosis was 160.6. Mean level of IgG was 255.69. CT / MRI finding of ring enhancing lesion or cerebritis was seen in 79 % of the cases with 18% of lesions in both basal ganglia and parietal lobes. Cerebritis was most common lesion in CT/MRI, seen in 16 cases while ring enhancing lesions were seen in 10 cases. 82% improved with treatment and 18% expired.

## Conclusions

The possibility of cerebral toxoplasmosis should be considered in every HIV-positive patient with neurological symptoms. In our study, Toxoplasmosis occured at CD4 levels >150, which should warrant prophylaxis for Toxoplasmosis at higher CD4 count. Parietal lobe lesions were common in our study, contrary to other existing data which say toxoplasma lesions are usually midline lesions.

